# Early clinical experience with a novel automated fecal management system for fecal incontinence in high-risk oncology and intensive care unit patients: a retrospective analysis and structured narrative literature review

**DOI:** 10.3389/fonc.2026.1895799

**Published:** 2026-10-07

**Authors:** Karen Lou Kennedy-Evans, Deanna Vargo

**Affiliations:** 1 Independent Researcher, Tucson, AZ, United States; 2 Independent Researcher, Cuyahoga Falls, OH, United States

**Keywords:** automated stool management, chemotherapy-induced diarrhea, fecal incontinence, indwelling balloon catheter, oncology intensive care, pressure-free fecal management, qoramatic, thrombocytopenia

## Abstract

**Background:**

Fecal incontinence (FI) is prevalent in critically ill oncology patients, frequently compounded by chemotherapy-induced diarrhea (CID) and severe thrombocytopenia. Conventional indwelling balloon catheters (IBCs), the standard approach to FI management in the ICU, generate intraluminal radial pressures that frequently exceed mucosal capillary perfusion thresholds and are formally contraindicated by manufacturers in patients with platelet counts below 50 x 10^9^/L. This retrospective analysis and structured narrative literature review evaluates the safety, fecal containment efficacy, and nursing workload impact of a novel pressure-free automated stool management (ASM) system in high-risk oncology ICU patients.

**Methods:**

A retrospective analysis was conducted at a major academic comprehensive cancer center in the United States during 2025. Outcome data from 190 unique adult oncology ICU patients, including two structured evaluation cohorts of 20 patients each, followed by broader institutional implementation (n=150), were compiled from standardized nursing quality improvement product evaluation forms completed throughout each patient’s device-use period. A prospectively designated thrombocytopenic subgroup (n=47; platelet counts <50 x 10^9^/L at device deployment) was analyzed separately. A structured narrative literature review was conducted across PubMed, MEDLINE, and CINAHL (final search: February 2026).

**Results:**

Across 190 unique device placements, zero device-related adverse events were observed (adverse event rate 0%; 95% CI upper bound 1.6%, rule of three). In the thrombocytopenic subgroup (n=47), zero device-related adverse events were observed (95% CI upper bound 6.4%). No device-related mucosal bleeding, perforation, ulceration, necrosis, or device malfunction requiring intervention was documented. The device achieved 100% successful fecal diversion, with no major leakage event (defined as stool output requiring linen replacement) recorded. The per-assessment leakage rate was 1.7% (11 positive assessments out of 646 total), all representing minor peripheral moisture requiring only topical hygiene management with no linen change. Daily nursing time with ASM was 6.8 minutes per patient per day; contextual comparison with published estimates of 120 minutes per patient per day (IBC protocol) and 174 minutes per patient per day (absorbent pad management) from Vargo et al. yields estimated differences of 94% and 96%, respectively, subject to methodological differences between datasets. Retrospective chart review identified no chemotherapy delays or ICU length of stay prolongation attributable to device-related complications.

**Conclusions:**

This early institutional experience with a novel pressure-free ASM across 190 unique oncology ICU placements, including 47 in patients with severe thrombocytopenia, was not associated with any observed device-related adverse event. These findings represent hypothesis-generating preliminary data and do not establish superiority over conventional devices. The clinical need for safe fecal management alternatives in thrombocytopenic oncology patients is substantive; these data provide rationale for appropriately powered prospective comparative trials.

## 1 Introduction

### 1.1 Epidemiologic context

Intensive care units (ICUs) in the United States manage approximately 5.7 million admissions annually (1). Among these, oncology patients constitute 15-20% of admissions at major academic medical centers, a proportion that has increased with the expanding use of intensive cancer-directed therapies including myelosuppressive chemotherapy, immune checkpoint inhibitors, and high-dose radiation protocols (2). Oncology ICU patients present with multiple overlapping physiologic vulnerabilities: underlying malignancy with associated multiorgan dysfunction, profound iatrogenic immunosuppression, and hematologic complications including thrombocytopenia, coagulopathy, and anemia. Within this context, fecal incontinence has emerged as a clinically significant yet frequently underrecognized complication with substantial direct and indirect morbidity.

### 1.2 Fecal incontinence in critically ill oncology patients

Fecal incontinence (FI), defined as involuntary leakage of stool or inability to maintain continence, affects 18-33% of general ICU patients, rising to 50-70% in those with moderate to severe diarrhea (3, 4). In oncology ICUs, prevalence is substantially higher due to chemotherapy-induced diarrhea (CID), radiation enteritis, and mucosal toxicities. High-grade (Grade 3-4) CID occurs in 10-32% of patients receiving irinotecan, 5-fluorouracil, and capecitabine-based regimens (5–7), 10-30% receiving immune checkpoint inhibitors (8), and 6-9% receiving EGFR-targeted agents (9). Pelvic and abdominal radiation adds acute and chronic motility and absorptive disturbances (10). CID episodes may persist 7–14 days or longer (6), creating sustained periods of anorectal stress that overwhelm continence mechanisms in already-compromised patients. In the subset with severe thrombocytopenia (platelet counts <50 x 10^9^/L), a common consequence of myelosuppressive regimens and hematologic malignancies, even minor mucosal trauma carries substantially amplified hemorrhagic risk (11, 12).

### 1.3 Clinical need and study objective

The standard approach to FI management in critically ill patients has relied on indwelling balloon catheters (IBCs), which achieve containment by inflating a retention balloon within the rectal lumen. These devices generate radial intraluminal pressures of 32–82 mmHg, routinely exceeding the mucosal capillary perfusion threshold of 25–30 mmHg and predisposing tissue to ischemic injury (13, 14). Published complication rates of 7-28% include mucosal bleeding, anal canal erosion, rectal necrosis, and perforation (15–18). Critically, IBC manufacturers explicitly contraindicate their use in patients with platelet counts below 50 x 10^9^/L on the basis of this risk profile, yet safe and effective alternatives for this population have until recently been lacking. Pharmacological agents such as octreotide have been used adjunctively (19), but do not address the mechanical containment challenge in bedridden patients.

This report describes the safety, fecal containment efficacy, and nursing workload impact of the Qoramatic Automated Stool Management System (Consure Medical), a novel pressure-free automated fecal management device, in high-risk oncology ICU patients at a major academic cancer center in the United States, including a pre-designated subgroup of 47 patients with severe thrombocytopenia in whom conventional IBCs are contraindicated.

## 2 Materials and methods

### 2.1 Study design and setting

This study presents a retrospective institutional analysis of a quality improvement evaluation combined with a structured narrative literature review. The clinical component was conducted at a major academic comprehensive cancer center in the United States during 2025. The institution operates dedicated oncology and hematology intensive care units staffed by oncology-certified registered nurses and critical care specialists. This analysis describes the retrospective review of outcome data collected prospectively through a formalized nursing quality improvement program evaluating an FDA *De Novo*-designated Class II medical device; it was not registered as a clinical trial. The institutional identity is withheld consistent with the blinded peer review process; the editorial office has been advised that the institution is an NCI-designated comprehensive cancer center, and institutional verification is available to the editors under confidentiality upon request.

### 2.2 Patient population and device placements

Adult patients (>=18 years) admitted to the oncology ICU in 2025 with confirmed fecal incontinence — defined as at least one documented episode of involuntary stool leakage within 24 hours prior to device deployment, who were fully bedridden were eligible for device use. The quality improvement evaluation comprised two sequential structured cohorts of 20 patients each (Cohort 1: n=20; Cohort 2: n=20; total n=40), followed by broader institutional implementation encompassing an additional 150 device deployments, yielding 190 unique patients in the complete series. Each patient received a single device placement; there were no repeated or duplicate placements. A prospectively designated clinical subgroup of 47 patients with severe thrombocytopenia (platelet counts <50 x 10^9^/L at device deployment) was identified on the basis of this platelet threshold, the clinical criterion that specifically informed the real-time decision to select the novel ASM over a conventional IBC, and is reported separately. All 47 thrombocytopenic patients are included within the total cohort of 190.

The cohort comprised adult patients with active oncological diagnoses requiring ICU-level care, encompassing hematologic malignancies (including acute leukemia, lymphoma, and myelodysplastic syndromes) and solid tumors (including gastrointestinal, thoracic, genitourinary, and other cancers). Based on the institutional case mix and the national prevalence of colorectal cancer as the third most common cancer diagnosis in the United States (20), approximately 20% of enrolled patients had colorectal malignancy, a population particularly susceptible to treatment-related CID from irinotecan- and fluoropyrimidine-based regimens (5, 6). Patient-level demographic data (age, sex, cancer stage, and individual laboratory values) were not systematically captured by the quality improvement evaluation forms, which were designed as clinical monitoring instruments rather than research data collection tools; this is acknowledged as a limitation.

### 2.3 Device description

The Qoramatic Automated Stool Management System (Consure Medical, Dallas, Texas, USA) holds both an FDA *De Novo* Designation (DEN250043/S003) and an FDA Device Listing. The device is indicated for fecal management in adult bedridden patients with liquid or semi-formed fecal incontinence, with a manufacturer-specified maximum use duration of 29 days per placement.

The core component is a soft, conforming silicone receptacle (biocompatibility evaluated per ISO 10993-1 (21)) that seats at the anal verge without intraluminal insertion, applying zero radial pressure (0 mmHg). No retention balloon is inflated within the rectal lumen. A closed-loop system of automated negative suction actively diverts fecal effluent into a dual-chamber, antimicrobial-lined reservoir (capacity ~ 1.5 liters), eliminating passive pooling and stool-skin contact. An automated irrigation function maintains cleanliness of the receptacle interface. The 0 mmHg radial pressure is a design and engineering characteristic inherent to the device’s passive receptacle mechanism, verified under bench-top conditions and documented in the manufacturer’s design history file. It was not measured clinically in the present patient series.

Device deployment was performed by trained bedside registered nurses following institutional nursing education delivered prior to the evaluation period. The procedure involved positioning the silicone receptacle at the anal verge, external securing without balloon inflation, and connection to the automated suction and irrigation console. Mean device use per patient was approximately 8 days; no patient required device use beyond 15 days. Devices were removed upon clinical resolution of fecal incontinence, ICU transfer, or completion of the acute care episode.

### 2.4 Outcome assessment and safety surveillance

Outcomes were assessed prospectively by bedside oncology ICU registered nurses using standardized product evaluation forms completed for each patient throughout the device-use period. These forms were developed by the clinical team as internal quality improvement instruments prior to initiating the evaluation and documented at each nursing assessment: (1) containment status (any leakage defined as fecal material contacting patient skin, bed linen, or external device surfaces); (2) perianal skin integrity and any observable mucosal change; (3) nursing time per care interaction, recorded at the time of each episode; and (4) overall device performance observations. Assessments were completed at each nursing shift change for the full duration of each patient’s device use. Across all 190 patients, 646 total nursing assessments were documented and constitute the denominator for the per-assessment leakage rate.

These product evaluation forms were internal quality improvement instruments only. They were not shared with the device manufacturer, were not designed as research data collection tools, and the manufacturer played no role in form design, data collection, analysis, or interpretation of findings.

Safety outcome definitions applied uniformly throughout this analysis: Device-associated mucosal bleeding was defined as visible fresh blood in the collection system or perianal region not attributable to the underlying malignancy or pre-existing anorectal pathology. Rectal perforation was defined as radiographic or surgical confirmation of full-thickness bowel wall disruption occurring during or attributable to device use. Ulceration or necrosis was defined as visible mucosal breakdown or tissue necrosis documented at device removal or nursing assessment. Overall device-related adverse event was defined as any of the above, or any device-related complication requiring unplanned medical or nursing intervention. Secondary outcomes included: fecal containment efficacy, assessed using a two-tier leakage classification — major leakage (stool output sufficient to require linen replacement, classified as a containment failure) and minor leakage (low-volume peripheral moisture at the anal margin requiring only topical hygiene management, no linen change). Successful fecal diversion was defined as the absence of any major leakage event during the patient’s device-use period. Leakage rate was reported as the proportion of individual nursing assessments with any documented leakage, major or minor. Additional secondary outcomes included daily nursing time per patient, ICU length of stay attributable to device-related complications, and chemotherapy continuity.

### 2.5 Statistical analysis

Outcomes are reported using descriptive statistics. No inferential statistical testing was performed given the retrospective observational design and absence of a contemporaneous comparator group. For zero-event proportions, two-sided 95% confidence interval upper bounds were calculated using the rule of three approximation (upper bound = 3/n), which provides a conservative estimate of the upper confidence limit when no events are observed (22). These interval estimates are explicitly reported to qualify the precision of zero-event observations and do not establish that the true event rate equals zero. Results are contextualized against published reference rates identified in the structured narrative literature review; these contextual comparisons are not direct comparative effectiveness estimates.

### 2.6 Structured narrative literature review

A structured narrative literature review was conducted across PubMed, MEDLINE, and CINAHL from 2000 to 2025 (final search: February 2026). Primary search terms were applied individually and in Boolean combination: fecal incontinence; indwelling balloon catheter; fecal management system; chemotherapy-induced diarrhea; thrombocytopenia AND oncology ICU; mucosal injury; rectal catheter complications; automated stool management. English-language peer-reviewed original research, systematic reviews, and clinical practice guidelines were prioritized. Reference lists of retrieved articles were hand-searched for additional relevant sources. Narrative synthesis focused on the pathophysiology of fecal incontinence in oncology populations, documented complications of conventional IBC use, and mechanistic rationale for pressure-free fecal management alternatives. This is explicitly a structured narrative review, not a systematic review or meta-analysis, and PRISMA reporting guidelines, which apply specifically to systematic reviews, are not applicable to this component.

### 2.7 Ethical considerations

This retrospective analysis was conducted under the quality improvement program of a major academic comprehensive cancer center in the United States. Data collection was performed prospectively using standardized nursing product evaluation forms as part of routine institutional quality improvement monitoring of a commercially available, FDA cleared Class II medical device. The retrospective review and analysis of fully de-identified aggregate outcome data collected through this quality improvement framework was determined by the institution’s clinical and nursing team not to constitute human subjects research; formal Institutional Review Board oversight was therefore not required. The study was conducted in accordance with the principles of the Declaration of Helsinki. No patient-identifying information was collected, retained, or included at any stage of this analysis. Individual patient consent was not required. The institutional identity is withheld consistent with the blinded peer review process; institutional verification is available to the editors under confidentiality upon request.

## 3 Results

### 3.1 Patient population

The complete series comprised 190 unique adult oncology ICU patients evaluated at a major academic cancer center in 2025. All 190 patients had confirmed fecal incontinence within 24 hours of device deployment and were fully bedridden; each received a single device placement with no repeated placements. The initial structured quality improvement evaluation comprised two sequential cohorts (Cohort 1: n=20; Cohort 2: n=20), followed by broader institutional deployment (n=150). Within the full cohort, 47 patients (24.7%) had documented platelet counts below 50 x 10^9^/L at device deployment and constitute the prospectively designated thrombocytopenic subgroup. The malignancy distribution was consistent with the case mix of a major U.S. academic cancer center; approximately 20% of patients had colorectal malignancy (20). Patient-level demographic data were not systematically captured by the quality improvement evaluation instruments; population characteristics are described at the cohort level (**Table 1**). Mean device use per patient was approximately 8 days (maximum: 15 days).

**Table 1:** Cohort structure and population characteristics.

Characteristic	Full cohort (n=190)	Thrombocytopenic subgroup (n=47)
Study period	2025	2025
Total unique patients	190	47 (within full cohort)
Cohort 1 (structured evaluation)	n=20	Included within total
Cohort 2 (structured evaluation)	n=20	Included within total
Broader institutional implementation	n=150	Included within total
Placements per patient	1 (no repeated placements)	1
Age eligibility	≥18 years (adult)	≥18 years (adult)
Mobility status	Fully bedridden	Fully bedridden
FI eligibility criterion	≥1 episode within 24h prior to deployment	≥1 episode within 24h prior to deployment
Platelet criterion	Not systematically captured (QI instrument)	<50 × 10^9^/L at deployment
Malignancy distribution (approximate)	Hematologic + solid tumors; ~20% colorectal	Predominantly hematologic; myelosuppressive regimens
Mean device duration	~8 days (maximum 15 days)	~8 days (maximum 15 days)

FI, fecal incontinence; QI, quality improvement. Patient-level demographic data (age, sex, cancer stage, individual laboratory values) were not systematically captured by the QI evaluation forms, which were designed as clinical monitoring instruments. Thrombocytopenic subgroup (n=47) is included within the full cohort (n=190). Colorectal proportion estimated from institutional case mix; consistent with Siegel et al. (20).

### 3.2 Safety outcomes

No device-related adverse events were observed across all 190 unique device placements. Zero instances of device-associated mucosal bleeding, rectal perforation, ulceration or necrosis, or device malfunction requiring unplanned clinical intervention were documented in either the full cohort or the thrombocytopenic subgroup. The overall device-related adverse event rate was 0% in both groups.

Applying the rule of three approximation for zero-event proportions (22): in the full cohort (n=190), the upper bound of the two-sided 95% CI for any device-related adverse event is 1.6% (3/190). In the thrombocytopenic subgroup (n=47), the upper bound is 6.4% (3/47). These interval estimates are reported to explicitly qualify the precision of zero-event observations; they do not imply that the true adverse event rate is zero, but rather represent a preliminary safety signal consistent with the device’s pressure-free design rationale.

Retrospective chart review identified no ICU length of stay prolongation and no chemotherapy delays or dose modifications attributable to device-related complications in any patient across the evaluation period. No failed insertions or placement failures requiring reinsertion were recorded. **Table 2** presents complete safety and efficacy outcomes.

**Table 2:** Safety and efficacy outcomes.

Outcome	Full cohort (n=190)	Thrombocytopenic subgroup (n=47)
Safety outcomes		
Device-related adverse events (any)	**0 (0%; 95% CI 0–1.6%)**	**0 (0%; 95% CI 0–6.4%)**
Device-associated mucosal bleeding	0	0
Rectal perforation	0	0
Ulceration or necrosis at device removal	0	0
Device malfunction (unplanned intervention)	0	0
Failed insertion or reinsertion required	0	0
ICU stay prolongation (device-related)	0	0
Chemotherapy delay attributable to device	0	0
Fecal containment efficacy		
Successful fecal diversion (patient-level)	**100%**	**100%**
Leakage rate (per nursing assessment)	1.7% (11/646 assessments)	—
Total nursing assessments documented	646	Included above

95% CI upper bounds calculated using the rule of three (3/n): Hanley and Lippman-Hand (22). Leakage defined as fecal material contacting patient skin, bed linen, or external device surfaces as documented by bedside nurses at routine assessment. Successful diversion defined as the absence of any major leakage event — defined as stool output sufficient to require linen replacement — during the patient’s device-use period. Minor leakage events (low-volume peripheral moisture not requiring linen change) do not constitute diversion failure and are reported separately as the per-assessment leakage rate. —, not separately reported for subgroup. Thrombocytopenic subgroup included within full cohort totals. Bold values indicate the primary composite safety outcome (device-related adverse events, any) for each study population.

### 3.3 Fecal containment efficacy

The device achieved 100% successful fecal diversion, defined as the absence of any major leakage event (stool output sufficient to require linen replacement) during the patient’s device-use period, in all 190 enrolled patients. The per-assessment leakage rate was 1.7%, representing 11 leakage-positive nursing assessments out of 646 total documented assessments. All 11 represented minor leakage — low-volume peripheral moisture at the anal margin requiring only topical hygiene management; no linen change was required in any instance. Leakage episodes were recorded by bedside nurses during routine shift-change assessments.

### 3.4 Nursing workload and operational impact

Device use was associated with a substantial reduction in nursing time allocated to fecal management. Mean daily nursing time per patient for device-related care was 6.8 minutes with the novel ASM, compared to operational reference figures of 120 minutes per patient per day for IBC management and 174 minutes per patient per day for absorbent pad and brief management — representing contextually estimated differences of 94% and 96%, respectively, relative to published reference figures reported in the peer-reviewed health economic analysis by Vargo et al. (23); these comparisons are drawn from different datasets and should not be interpreted as directly measured treatment effects. **Table 3** presents the nursing workload and cost comparison. Device acquisition and consumable supply costs are included in the total per-patient-day cost for the ASM arm, consistent with the fully-loaded cost methodology of Vargo et al. (23).

**Table 3:** Daily nursing workload and labor cost comparison for fecal incontinence management in the oncology ICU.

Management approach	Nursing time (min/patient/day)	Nursing labor cost^a^ (USD/patient/day)	Notes
Absorbent pads and briefs	174	$130.50	Includes repositioning, linen changes, skin care, hygiene per stool episode (23)
Conventional IBC	120	$90.00	Includes monitoring, balloon inflation checks, complication surveillance (23)
**Qoramatic ASM (this series)**	**6.8**	**$5.10** (**labor only)**^b^	Device and consumable supply costs included in total per-patient-day cost (not reflected in labor column)

^a^ Nursing labor calculated at USD $45.00/hour for registered nursing time (23, 24). Patient care attendant rate (USD $20.00/hour) applied to ancillary activities and not separately itemized here.

^b^ Labor cost for Qoramatic ASM reflects nursing time only; total per-patient-day cost including device acquisition and supplies is reported by Vargo et al. (23) as USD $90–131 versus $444–1,520 for conventional management, inclusive of all device and labor components. IBC, indwelling balloon catheter; ASM, automated stool management. Bold values indicate the study device (Qoramatic ASM) and its observed outcomes as reported in the present series.

## 4 Discussion

### 4.1 Pathophysiology of fecal incontinence in oncology ICU patients

Fecal continence depends on integrated function of anorectal anatomy, neuromuscular coordination, and cognitive awareness (25, 26). In oncology ICU patients, this system is disrupted at multiple levels simultaneously. Chemotherapy-induced diarrhea is the primary driver: irinotecan produces delayed diarrhea through enterohepatic recirculation of its active metabolite SN-38 and mucosal apoptosis (27, 28); bolus 5-fluorouracil causes Grade 3–4 diarrhea in approximately 32% of recipients via thymidylate synthase inhibition (7); capecitabine and other fluoropyrimidines produce similar effects in 10-20% of patients (29); immune checkpoint inhibitors trigger immune-mediated colitis in 10-30% (8, 30); and EGFR-targeted agents induce secretory diarrhea in 6-9% (27). Pelvic radiation adds acute and chronic enteritis with motility and absorptive disturbances (10). CID episodes may persist 7–14 days or longer (6, 19), creating sustained anorectal stress in patients whose mucosal defenses are simultaneously compromised by treatment-related toxicity.

Chemotherapy and radiation render anorectal mucosa inherently vulnerable through epithelial apoptosis, impaired tight junctions, reduced mucin production, microvascular compromise, and delayed tissue regeneration (31), a tissue environment poorly tolerant of additional mechanical insult. When combined with severe thrombocytopenia (platelet counts <50 x 10^9^/L), common with myelosuppressive regimens and hematologic malignancies, even minor mucosal trauma carries substantially amplified hemorrhagic risk through impaired primary hemostasis, coagulation dysfunction, and the synergistic interaction between pressure-induced ischemia and reduced platelet reserves (11, 12, 32). The prevalence of FI in oncology ICU patients can reach 40-50% during acute treatment phases (33), substantially exceeding general ICU rates.

### 4.2 Limitations of conventional indwelling balloon catheters: clinical context

The complication profile of conventional IBCs in the published literature provides the clinical context motivating this evaluation. IBCs generate radial intraluminal pressures of 32–82 mmHg through retention balloon inflation, routinely exceeding the mucosal capillary perfusion threshold of 25–30 mmHg and predisposing tissue to ischemia, progressive epithelial necrosis, and ulceration (13, 14). In high-risk ICU cohorts, published complication rates include mucosal bleeding in 5-15% of patients, anal canal erosion in 8-10%, and rectal necrosis in 2-5% (15, 16, 18). Pittman et al. (17) specifically reported adverse outcomes with conventional IBC use in patients with severe thrombocytopenia; IBC manufacturers formally contraindicate their devices in patients with platelet counts below 50 x 10^9^/L on this basis (34). The FDA Manufacturer and User Facility Experience (MAUDE) database documents over 450 reports of bleeding or trauma and over 90 overinflation events associated with balloon catheter use from 2015 to 2025 (35); these report counts are not incidence estimates and cannot be used as such without validated exposure denominators, but provide regulatory signal of the population-level scale of this problem.

These literature-reported complication rates are presented solely as the clinical context motivating the evaluation of a pressure-free alternative, not as direct comparators to the institutional data reported here. Population heterogeneity, surveillance methodology differences, and variation in outcome definitions preclude direct comparison, and no comparative effectiveness conclusion is intended or drawn.

### 4.3 Pressure-free design: mechanistic rationale for the observed safety profile

The pressure-free design of the Qoramatic ASM eliminates the primary mechanism of IBC-related mucosal injury by removing intraluminal balloon pressure entirely. By seating at the anal verge without intraluminal insertion, the silicone receptacle applies zero radial pressure (0 mmHg), a design characteristic confirmed through bench testing. Active fecal diversion through automated negative suction addresses a well-documented failure mode of passive balloon systems during high-volume or high-viscosity CID episodes. The closed dual-chamber reservoir minimizes clinician stool exposure and reduces cross-contamination risk in the profoundly immunosuppressed oncology ICU environment (36–39).

The observation of zero clinically apparent device-related adverse events across 190 placements is mechanistically consistent with this pressure-free design rationale. It is explicitly noted, however, that bench-validated design characteristics and clinical observational findings are distinct lines of evidence; the absence of observed complications in a nursing-surveillance-based series does not exclude the possibility of subclinical mucosal changes below the clinical detection threshold of shift-based observational monitoring. This distinction is addressed in the Limitations section.

### 4.4 Clinical significance and implications

The contextually estimated 94–96% difference in daily nursing time for fecal management carries significant operational implications for oncology ICU settings. The reallocation of nursing attention from routine fecal management to higher-complexity clinical activities may have unmeasured secondary benefits for overall care quality in an environment where nursing time is a finite critical resource. These findings are consistent with the peer-reviewed health economic analysis by Vargo et al. (23), which reported total per-patient-day costs of USD $90–131 for novel ASM management compared to USD $444-1,520 for conventional management, inclusive of device, supply, and labor components. The convergence between the institutional nursing time observations reported here and the independently derived economic data of Vargo et al. (23) provides contextual support, while acknowledging the limitations of both datasets.

The economic burden of FI complications in the oncology ICU extends beyond device costs. Each IBC-related complication event is estimated to prolong ICU length of stay by 3–7 days, generating incremental costs of $10,000-$20,000 per event (14, 17). In a population where treatment continuity directly affects cancer outcomes, device-related complications that delay chemotherapy carry clinical consequences disproportionate to their direct cost. Retrospective chart review of this series identified no ICU length of stay prolongation and no chemotherapy delays attributable to device-related complications — a secondary observation consistent with the safety profile, though not formally testable in an observational design without a comparator group.

The specific clinical need in thrombocytopenic oncology patients warrants particular emphasis. Patients with platelet counts below 50 x 10^9^/L who develop severe FI during oncology ICU care currently face a clinical dilemma: conventional IBCs are contraindicated, while absorbent pad management is associated with persistent stool-skin contact, perianal skin breakdown, infection risk, and the highest nursing burden. The observation of zero device-related adverse events in 47 such patients across this evaluation provides clinically meaningful hypothesis-generating data for prospective investigation in this underserved population.

### 4.5 Relevance to colorectal cancer and the gastrointestinal oncology population

Patients with colorectal cancer are disproportionately affected by treatment-related diarrhea: irinotecan, 5-fluorouracil, capecitabine, and pelvic radiation, the therapeutic backbone of CRC management, are among the highest-risk agents for high-grade CID (5–7, 40). The anorectal mucosal fragility associated with these regimens, combined with the hemorrhagic risk amplification from thrombocytopenia in patients receiving myelosuppressive CRC chemotherapy, makes this population a clinically compelling use case for pressure-free fecal management. With approximately 20% of the institutional cohort having colorectal malignancy (20), consistent with colorectal cancer’s status as the third most common cancer in the United States, the findings reported here have direct and specific relevance to the gastrointestinal oncology population. The maintenance of chemotherapy continuity, with zero observed treatment delays attributable to device-related complications, is particularly consequential in CRC, where schedule adherence is a determinant of treatment outcomes (41).

## 5 Limitations

This study carries several limitations that should inform interpretation of its findings. First, the retrospective design and reliance on nursing-completed quality improvement forms, rather than a prospective research protocol with pre-specified endpoints and independent outcome adjudication, introduces the possibility of incomplete outcome capture and limits the reproducibility of the ascertainment methodology. Second, the absence of a contemporaneous institutional comparator group means that all contextual comparisons with conventional IBC outcomes are drawn from published literature rather than directly observed paired experience and are subject to well-recognized confounders of population heterogeneity, surveillance intensity, and outcome definition differences. No comparative effectiveness conclusions are drawn from this study.

Third, while the complete institutional series comprises 190 unique patients, the formal structured evaluation cohorts (n=40) are modest in size, and the broader implementation phase (n=150) reflects real-world deployment rather than a controlled evaluation. This is a single-institution series from a major academic cancer center; outcomes may not generalize to institutions with different staffing models, patient acuity levels, or nursing training programs. Fourth, outcome ascertainment for safety events was based on nursing observational documentation rather than scheduled endoscopic surveillance. Subclinical mucosal changes not producing clinically observable signs would not have been captured by this protocol. The reported zero adverse event rate should therefore be interpreted as “zero clinically apparent device-related complications detected by the applied nursing observation protocol” — a meaningful finding that does not exclude biological-level effects below the clinical detection threshold.

Regarding the thrombocytopenic subgroup specifically: n=47 is modest by the standards of prospective controlled trials. However, within the specific clinical context of this subgroup, patients in whom conventional IBCs are formally contraindicated and in whom any mucosal trauma carries amplified hemorrhagic risk,the observation of zero device-related adverse events across 47 placements carries meaningful clinical signal, particularly given that the upper bound of the two-sided 95% confidence interval (6.4%) excludes complication rates comparable to the lower end of the published IBC range (7-28%). This observation becomes more clinically interpretable when considered alongside the manufacturer’s independently gathered real-world evidence dataset of more than 4,000 patients across diverse U.S. ICU settings (23) — the two datasets are not formally combined, but the larger dataset demonstrates that the safety profile observed in this series is not an isolated institutional finding.

Fifth, individual-level demographic and laboratory data, including age, sex, cancer stage, specific platelet values, comorbidity profiles, and anticoagulation status — were not captured in the quality improvement evaluation forms. This limits the characterization of the patient population and is acknowledged as a material gap for a research publication; it is the most significant methodological limitation of repurposing QI documentation for peer-reviewed reporting. Sixth, nursing time estimates were derived from care-episode timing recorded on evaluation forms rather than from formal concurrent time-and-motion study methodology and should be considered structured operational estimates rather than rigorously validated time-motion data. Finally, device duration data (mean approximately 8 days; maximum 15 days) represent institutional operational estimates and individual variation within the broader implementation phase may not be precisely captured.

## 6 Conclusions

Fecal incontinence in critically ill oncology patients, particularly those with chemotherapy-induced diarrhea and severe thrombocytopenia, represents a substantive clinical and operational burden for which conventional indwelling balloon catheters carry well-documented mucosal injury risks and, in thrombocytopenic populations, formal manufacturer contraindications. This early institutional experience with a novel pressure-free automated stool management system across 190 unique oncology ICU placements, including 47 in patients with platelet counts below 50 x 10^9^/L, was not associated with any observed device-related adverse event. The upper bound of the two-sided 95% confidence interval for device-related adverse events was 1.6% in the full cohort and 6.4% in the thrombocytopenic subgroup, appropriately qualifying what zero observed events establishes at these sample sizes. Nursing workload with ASM was substantially lower than published conventional management benchmarks, with a contextually estimated difference of 94–96% relative to reference figures from Vargo et al.; this comparison is drawn across different datasets and should be interpreted accordingly.

These findings represent hypothesis-generating preliminary data from a single institution and do not establish comparative superiority over conventional fecal management devices, eliminate the possibility of adverse events at higher statistical precision, or support modifications to clinical practice guidelines without prospective comparative validation. The pressure-free mechanism of action provides a coherent biological rationale for the observed safety profile, but bench-validated design characteristics and clinical observational findings are distinct lines of evidence that must not be conflated. The clinical urgency of identifying safe and effective fecal management options for thrombocytopenic oncology patients is substantial; the current standard — a contraindicated device or absorbent pad management with its attendant skin injury risk and nursing burden — is inadequate. These preliminary data provide sufficient rationale for the conduct of appropriately powered, prospective, controlled trials in oncology ICU populations, with particular priority given to patients with severe thrombocytopenia and high-grade chemotherapy-induced diarrhea.

## Data Availability

The original contributions presented in the study are included in the article/supplementary material, further inquiries can be directed to the corresponding author.
